# Wastewater-Based Epidemiology as an Early Warning System for the Spreading of SARS-CoV-2 and Its Mutations in the Population

**DOI:** 10.3390/ijerph18115629

**Published:** 2021-05-25

**Authors:** Tomáš Mackuľak, Miroslav Gál, Viera Špalková, Miroslav Fehér, Katarína Briestenská, Miriam Mikušová, Karolína Tomčíková, Michal Tamáš, Andrea Butor Škulcová

**Affiliations:** 1 Department of Environmental Engineering, Institute of Chemical and Environmental Engineering, Faculty of Chemical and Food Technology, Slovak University of Technology, Radlinského 9, 812 37 Bratislava, Slovakia; miroslav.feher@stuba.sk (M.F.); mike.tamas@gmail.com (M.T.); xskulcova@stuba.sk (A.B.Š.); 2 Department of Inorganic Technology, Faculty of Chemical and Food Technology, Slovak University of Technology, Radlinského 9, 812 37 Bratislava, Slovakia; miroslav.gal@stuba.sk (M.G.); viera.spalkova@stuba.sk (V.Š.); 3 Department of Zoology and Fisheries, Faculty of Agrobiology Food and Natural Resources, Czech University of Life Sciences Prague, Kamýcká 129, 165 21 Prague, Czech Republic; 4 Biomedical Research Center of the Slovak Academy of Sciences, Institute of Virology, Dúbravská cesta 9, 845 05 Bratislava, Slovakia; katarina.briestenska@savba.sk (K.B.); miriam.mikusova@savba.sk (M.M.); karolina.tomcikova@savba.sk (K.T.)

**Keywords:** wastewater monitoring, SARS-CoV-2, COVID-19, PCR methods, biosensors, virus detection, genetic sequencing

## Abstract

New methodologies based on the principle of “sewage epidemiology” have been successfully applied before in the detection of illegal drugs. The study describes the idea of early detection of a virus, e.g., SARS-CoV-2, in wastewater in order to focus on the area of virus occurrence and supplement the results obtained from clinical examination. By monitoring temporal variation in viral loads in wastewater in combination with other analysis, a virus outbreak can be detected and its spread can be suppressed early. The use of biosensors for virus detection also seems to be an interesting application. Biosensors are highly sensitive, selective, and portable and offer a way for fast analysis. This manuscript provides an overview of the current situation in the area of wastewater analysis, including genetic sequencing regarding viral detection and the technological solution of an early warning system for wastewater monitoring based on biosensors.

## 1. Introduction

The idea of determining the consumption of illicit drugs on the basis of analysis of wastewater was introduced by Daughton and Ternes already in 1999 [1]. The development of new methodologies and advances in analytical chemistry has gradually led to the emergence of the term “sewage epidemiology” in the scientific area. In his study in 2005, Zuccato pointed to the possibility of analyzing illicit drugs in wastewater, putting the Daughton and Ternes hypothesis into practice for the first time [2]. He gradually found that based on the analysis of the dominant drug metabolite it is possible to estimate the number of doses used at the selected sewerage area. After 15 years, it can be stated that this scientific field is constantly evolving and progressing. This is evidenced by studies from 2019 and 2020, which describe the possibilities of monitoring various groups of endogenous human metabolism biomarkers, new types of drugs and psychoactive substances (NPS), the issue of pesticide and mycotoxin contamination of food and drinking water, or the possibility of identifying resistance genes for a selected antibiotic in the population under review [3,4,5,6,7]. For example, based on the quantitative measurement of specific biomarkers in wastewater from different regions and cities, we can subsequently evaluate the lifestyle of a particular population, the incidence of certain types of diseases, as well as the negative or positive impacts of the environment on its health [8]. However, it should be noted that the monitoring of the health status of a population in a given region may not always be welcome. It can point to environmental pollution in a given area and its subsequent impact on a region or a certain population, which may ultimately have an impact, for example, on the tourism, real estate market, and the like.

It can be said that the development of wastewater monitoring is mainly due to research activities taking place in Europe. In 2013, the COST ES1307 project was launched under the auspices of the EU Horizon 2020 Framework Program, bringing together several experts in the field of epidemiology and drugs and pharmaceuticals analysis in wastewater. Wastewater = based epidemiology connected with early detection in the case of a pandemic situation is just the next step in public health control [7].

## 2. Coronavirus SARS-CoV-2 and Wastewater

Viruses, even if we have an increasingly advanced healthcare system, may cause a wide range of health problems in the population, raising considerable concern because of their ability to mutate and often resist various disinfection procedures [9]. Many viruses cause severe or fatal diseases in humans. Examples include hemorrhagic fever caused by viruses such as Ebola or Marburg with a mortality rate above 60%, or rabies caused by RNA viruses of the genus *Lyssavirus* (mortality if symptoms develop is above 99.9%) [10,11]. Some rapidly spreading seasonal viruses can cause considerable economic damage in the form of reduced labor productivity, in addition to increased healthcare costs. An example may be influenza virus genetic mutations that have caused multiple epidemics. To a lesser extent, local epidemics have been observed for the coronaviruses MERS-CoV and SARS-CoV [12]. However, at the beginning of 2020, the SARS-CoV-2 coronavirus caused a worldwide pandemic of COVID-19, which will severely affect the economy of virtually all countries in the world [13].

The size of viruses (in tens or hundreds of nanometers) allows them to be easily transported in various environmental compartments (often adsorbed on small particles) [9]. In the first part of our review, we will focus on the possibilities of survival of the SARS-CoV-2 virus in different environments and on different surfaces. The study of Holbrook et al. (2020) shows that SARS-CoV-2 can be identified for a certain period of time on different surfaces—SARS-CoV-2 can be identified on plastics and steel even after 72 h, on paper after 24 h, on copper after 4 hours, and in aerosol particles after 3 h [14]. It has also been found that approximately 2–49.5% of COVID-19 positive cases had diarrhea [15,16,17,18] and 3.6–66.7% had vomiting, with RNA virus being identified in stool [17,18], but the WHO currently does not record any transmission of COVID-19 via the oral–fecal route [17]. On the contrary, the study published by Yeo et al. (2020) confirmed the possible transmission of SARS-CoV-2 by the oral–fecal route due to evidence of the aerosol transmission route during the outbreak of SARS in 2003 [19,20]. These inconsistent statements are due to the fact that not so much data is currently available. It is still not entirely clear why some individuals show stronger immune responses to the virus than others. Further data obtained in the coming years will clarify these deviations and differences. A study published by Medema et al. (2020) also points out the possibility of virus detection in wastewater by RT-PCR analysis [21]. In identifying the virus, the authors of the study focused on the detection of three fragments of the gene (*N1–N3*) of the nucleocapsid protein and one fragment of the gene (*E*) of the envelope protein as evidence of the presence of the virus. This finding is important for the implementation of wastewater monitoring as a tool to monitor the prevalence of SARS-CoV-2 in the population [21,22]. A study from Helsinki (Finland) describes the stability of RNA copy numbers of SARS-CoV-2 in wastewater regarding storage conditions. RNA counts remained surprisingly stable at 4 °C, −20 °C, and −75 °C after 29, 64, and 84 days. In conclusion, freezing temperatures should be used for storage of wastewater in order to conduct SARS-CoV-2 RNA analysis [23]. In order to obtain adequate monitoring data, in addition to detecting the presence of the virus in the wastewater, it is necessary to know the approximate percentage of people who excrete virus fragments or the virus itself in excrements. In Pan et al. (2020), it was found that virus excretion was present in nine out of seventeen infected cases [24]. Woelfel et al. (2020) describe the incidence in eight out of nine infected [25]. Chen et al. (2020) describe the presence of RNA in 12 out of 22 infected [26]. Lescure et al. (2020) describe the presence of RNA in excrement in two out of five infected [27]. Wang et al. (2020) and Woelfel et al. (2020) attempted to cultivate the virus from excrement [25,28]. Wang et al. (2020) were successful and were able to cultivate the active virus twice from four samples [28]. Woelfel et al. (2020), with thirteen samples, were not successful at all [25]. Another important factor is the different duration of the virus release from an infected individual in feces [29,30]. However, it is also necessary to know the extent of virus release during infection from the human body (some viruses that can spread well by water are released from the human body in an amount of 10^2^ to 10^12^ per gram of excrement) [9]. Pan et al. (2020) found a load of SASR-CoV-2 in excrement from 550 copies to ~10^5^ copies per mL [24]. Lescure et al. (2020) describes the incidence of ~10^6^–10^8^ copies in one patient [27]. Woelfel et al. (2020) observed the occurrence of the RNA virus in eight different patients and found that in one patient the RNA copy value could reach ~10^8^ per oro- or nasopharyngeal swab during peak infection [25]. Randazzo et al. (2020a) found SARS-CoV-2 RNA titers in untreated wastewater samples of ~10^2^ genomic copies per mL, while tertiary treated water was negative [31]. On the contrary, Haramoto et al. (2020) analyzed wastewater influent and treated effluent before the chlorination and in case of large number of infected cases was effluent positive for the virus presence. [32]. The authors pointed out on the early detection in wastewater (stools) compared to reported cases. Overall, the results of wastewater monitoring in Spain, Brazil, and Italy show that SARS-CoV-2 was circulating in the country since end of the November 2019, much earlier than the first reported cases in these countries [33,34,35].

Li et al. (2020) point out in their study that as many as 86% of individuals infected with the virus were asymptomatic, and therefore wastewater monitoring could also be one of the important indicators of disease prevalence in a city or region [36]. Wastewater from healthcare facilities (especially from hospitals with a higher incidence of COVID-19 patients) may have an increased occurrence of this virus in the wastewater produced. Additionally, the persistence of SARS-CoV-2 in wastewater and the mechanisms of its inactivation such as wastewater treatment, UV, or disinfection should be investigated, regarding direct effects on the viral fragment quantity [37,38]. The persistence of SARS-CoV-2 infectivity vs. the RNA signal in wastewater was evaluated in Bivins et al. (2020) and the authors found that infectious virus was not as persistent in wastewater as viral RNA, which indicates that detection of viral RNA in wastewater does not substantiate the risk of infection [39]. Similarly, the potential for infection due to the contact with wastewater by the present viral RNA is negligible [40,41]. Additionally, wastewater-based epidemiology also seems to be the only viable means of effective surveillance for poor regions and nations [42].

### Wastewater—A Possible Source of Information

The emergence of infectious outbreaks, local epidemics, and global pandemics is difficult to predict. This is where we show our significant vulnerability—a lack of opportunity to respond in time. This is due to the established practices of classical epidemiology, which only identifies the outbreak of a disease based on certain clinical manifestations [9]. In this section, we will focus on whether wastewater can be used as a source of important information on the spread of COVID-19 in the population.

The prevalence of certain types of viruses in wastewater may vary significantly during the year. The study by Katayama et al. (2008) stresses that the occurrence of noroviruses in wastewater is most pronounced from November to April, while the adenovirus concentration can be considered constant throughout the year [43]. As already described, some groups of pathogenic viruses, e.g., noroviruses or enteroviruses are seasonal but can be detected in growth biofilms on the pipe walls throughout the year. Biofilm can thus contribute to their occurrence and spread in wastewater throughout the year [44]. The effect of climatic factors on the COVID-19 pandemic was described by Bashir et al. (2020) [45]. There was no evidence that warm weather can suppress actual pandemic.

Nowadays, there is a challenge for technologists and scientists from the field of wastewater monitoring, focused primarily on the possible detection and description of trends of diseases caused by viruses such as coronavirus SARS-CoV-2. Based on various studies, it is believed that the active SARS-CoV-2 virus or its degradation fragments can be delivered into wastewater via feces of infected patients [18,19,21]. It was even confirmed that fecal viral shedding was positive 1–33 days after negative test from nasopharyngeal swab [46]. It would be useful to consider the effectiveness and consequences of wastewater treatment and the possible subsequent spread of the virus into the environment [47]. If further studies from other parts of the world confirm the possible detection of the virus in wastewater, this methodology may detect a wave of spread of the infection or its possible return much faster and cheaper than testing of symptomatic individuals. In a paper by Wu et al. (2020b), the authors tested wastewater from urban WWTP in Massachusetts and found that the amount of SARS-CoV-2 in wastewater samples was significantly higher than was expected based on clinically-confirmed cases [48]. Unless further experiments are completed, the question remains whether the clinical estimates are correct. Monitoring of viruses in wastewater requires data describing their amount, which can be obtained by a quantitative polymerase chain reaction (referred to as qPCR or RT-PCR), as an increase in virus concentration indicates a possible onset of disease outbreak [9]. The RT-qPCR method was actually used in detection and quantification of SARS-CoV-2 RNA in wastewater from a WWTP in Australia. An analysis leading to two positive detections and an estimated RNA copy number was used to estimate the number of infected individuals. The estimated range of infected individuals correlated with clinical observations [49]. A similar experiment was carried out in Paris. Raw and treated wastewater were tested for the presence of SARS-CoV-2 RNA using RT-qPCR analysis. The wastewater was collected from three major WWTPs, to which 3–4 million people are connected. The results showed that the treated wastewater from WWTP effluent contained 100 times fewer genomes compared to raw water from influent. Additionally, the increase of SARS-CoV-2 genomes in raw wastewater correlated with the increase of fatal cases in Paris, and therefore this study demonstrates that the wastewater contamination occurred before the beginning of the exponential increase of the epidemic [50]. Kocamemi et al. (2020a) submitted information about SARS-CoV-2 RNA occurrence in wastewater treatment plants and manholes in Istanbul [51]. Five samples out of seven from WWTP tested positive as did all of the samples from manholes. Very interesting results were offered in a study by Rimoldi et al. (2020), where raw and treated wastewater from WWTP and one river in Milano were compared in order to detect SARS-CoV-2 virus presence and infectivity [52]. Samples of raw wastewater were positive to PCR amplification, but infectivity was not significant. Treated water was always negative, and some samples from the river were positive with PCR amplification. The vitality of viruses was negligible, indicating the absence of sanitary risks. Monitoring of SARS-CoV-2 or its fragments is currently realized in wastewater in the USA (Louisiana, Detroit), Czech Republic, Buenos Aires in Argentina, Frankfurt in Germany, or in sludge (Istanbul) [53,54,55,56,57,58]. However, there are not enough data available for the sludge contamination [20].

Monitoring of time changes in the occurrence of viruses in wastewater in combination with analysis of selected metabolites and biomarkers (creatinine, cholesterol, ammonia, nitrogen, and others) in municipal wastewater [9,59,60,61] or analysis of active sim cards (identity cards in mobile devices) [62] may lead to a description of the sites where the outbreaks arise. In addition, consistently selected sampling points from the sewage system may allow for the definition of sites or regions from where the disease can spread (so-called point-of-focus) [9]. Additionally, mathematical model of virus spread can be developed until the “patient zero” is found [63].

As with the monitoring of illicit drug use, there may be some obstacles and shortcomings in the monitoring of viruses in wastewater [64]. It is mainly the analytical complexity of the detection of the desired viruses, the possible inconsistency of ongoing sampling, and the subsequent analyses that require some investment and operating costs. Monitoring may also be complicated by the high proportion of ballast water and wastewater from healthcare facilities. It must be determined whether and to what extent virus monitoring is influenced by factors such as the composition of the microbial community in the monitored sewerage system, chemical pollution, type of sewerage system, residence time in the sewerage network, pH value, temperature, etc. [9,65].

For monitoring of certain diseases based on wastewater analysis to be successful on a larger geographical scale than at the national level, national authorities need to be aware of the potential benefits of this monitoring method, for example in controlling the spread of COVID-19 [64]. National grant agencies should provide increased support and development of different capacities, in particular in the form of grants related to wastewater monitoring at wastewater treatment plants. However, it should be noted that monitoring of the status and trends of the spread of COVID-19 will require some adjustments or changes compared to existing well-established practices and methods used to monitor, for example, drug use [64]. On the positive side, there is already an established network of scientific institutions and workplaces in several European countries for monitoring illicit drugs (transnational studies describing in particular the issue of drug use and consumption are regularly published) [66,67,68], which could also be used to create an early warning system for an upcoming pandemic.

Research in this field has now started intensively in several European countries (the Netherlands, Sweden, and Switzerland, but also in Slovakia) and the US, leading to the creation of a “Wastewater-Based Epidemiology for COVID-19” platform for rapid communication of professionals dealing with and the issue of SARS-CoV-2 in wastewater [69]. A summary of knowledge and critical factors for implementation of sewage epidemiology was described in Polo et al. (2020) [70].

Some important studies focused on WBE are summarized in Table 1.

Table summarizes target genes used for monitoring wastewater for SARS-CoV-2 in appropriate studies. The most frequently used primers target *N* genes, which are also recommended by the CDC [77]. RNA SARS-CoV-2 was detected in wastewater worldwide such as in Europe [21,23,31,32,41,50,52,74], Asia [32,55,71,72,73,75], America [48,76], and Australia [49].

It can be assumed that the issue of wastewater monitoring will gradually begin to interfere with other scientific disciplines, such as microsensorics and gradual automation of data collection. It is therefore necessary to wait and see what time (and wastewater) will bring us in this new wastewater monitoring industry. However, wastewater monitoring is a non-invasive monitoring method that does not burden the healthcare system and can be used for local monitoring of closed communities.

## 3. Detection of SARS-CoV-2

Viruses are infectious agents distributed worldwide, and thus they represent a great concern for human health. As we have shown in the previous sections, rapid and accurate detection of the virus in various environments is an important part of dealing with any minor or major epidemic.

### 3.1. Traditional (Standard) Techniques for Virus Detection

For detection of viruses in biological samples, several different techniques were developed [78,79]. Whole virions can be observed and identified directly under an electron microscope. However, this method is rarely used due to the complex process of sample preparation [80,81]. The most common are cell culture-based techniques and direct detection of viral proteins or nucleic acids [82]. These methods must be very specific and sensitive, because the concentration of the virus in environmental samples (especially from water) is usually very low [83,84].

**Figure 1:**
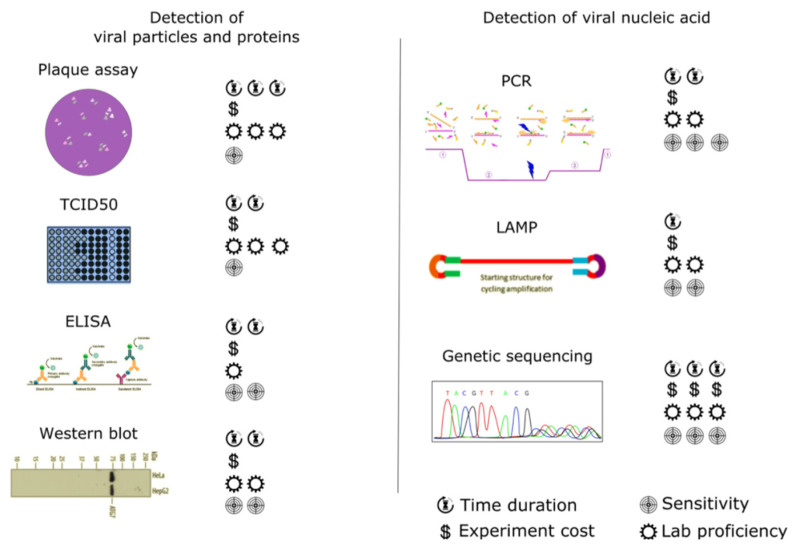
Comparison of currently used methods for the detection of viruses. The detection methods are divided into 2 subgroups differing in targeting various parts or molecules of the virus.

In this part of the review, we will briefly present experimental techniques that can be used for the detection of SARS-CoV-2 in wastewater (Figure 1). In the first part of this section, we will focus on the detection of viral particles and proteins, in the second part viral nucleic acids, and in the last part of this section we will briefly introduce the latest experimental techniques used in the detection of viruses.

#### 3.1.1. Detection of Viral Particles and Proteins

Cell culture-based techniques such as “tissue culture infectious dose-50” (TCID50) and “plaque assay” are applied as a gold standard for the quantification of infectious viruses in environmental samples [83,85]. “Immunofluorescence assay linking cell cultures and antibody detection” is a bit more sophisticated—the method is based on the specific reaction between viral protein present in the host cell and a specific antibody. The signal emitted by the detected antibody labelled with fluorescent dye (fluorescein isothiocyanate) is visualized under a special fluorescent microscope so that the fluorescent signal in different compartments of cells such as the nucleus, cytoplasm, or cell membrane can be observed [81]. A novel technique, called integrated cell culture PCR, combines cell-culture and PCR techniques for detection and quantification of viruses. By incorporating the benefits of both methods, it overcomes their drawbacks and provides increased sensitivity and faster detection. However, the techniques mentioned above are time-consuming, relatively expensive, and in addition, not all viruses produce clear cytopathic effects or plaques, and some viruses cannot even be cultivated in vitro [86,87].

Immunological assays using specific antibodies against the target antigen have been introduced but require a high concentration of virus in the material (10,000–100,000 viral particles per milliliter), which may be a problem in cases of environmental samples. Enzyme-linked immunosorbent assay (ELISA) is a simple and fast method that can be used for direct detection and quantification of viral proteins or whole viral particles [88,89]. There are many variants of antigen detection ELISA, but the principle is always the same—the target antigen is detected by colorimetric chromatic reaction after binding with specific antibodies. The first step is the immobilization of the target antigen on the surface of wells in 96-well specially-treated polystyrene plates. The next step is the direct binding of specific detection antibodies—primary antigen-specific antibody and secondary antibody conjugated with the enzyme horseradish peroxidase. The last step is the addition of the substrate (the most common is chromophore o-phenylenediamine with hydrogen peroxide) and spectrophotometric analysis of the chromatic reaction for quantitative representation of the target antigen [79,89,90,91,92]. An exception is the competitive ELISA, where labeled antigen is used instead of labeled antibody for generating a chromatic reaction. This labeled antigen competes with the target antigen for the binding site on a specific antibody. Therefore, the signal is inversely proportional to the amount of the target antigen. The specificity and sensitivity of ELISA variants depends on the selected antibodies. The most efficient is the sandwich ELISA, using two antibodies interacting with each other as a pair only in presence of the target antigen, but designing such antibodies is difficult [79,89,93].

The Western blot (WB) assay or immunoblotting is a common method used to study proteins [94]. It has a wide range of applications within biochemistry, physiology [95], virology, and microbiology and also in medicine as a confirmatory assay. The advantages of WB include sufficient sensitivity and specificity. The WB assay allows one to identify and analyze the target protein or quantify protein levels in the biological samples [96]. The protein structure depends on experimental conditions used during protein sample preparation—under non-denaturing conditions, the proteins maintain their native structure, while the denaturing conditions result in linearized proteins. The WB procedure involves the separation of proteins from a complex mixture, according to their molecular weight, by using polyacrylamide gel electrophoresis [97]. The separated molecules are then transferred (blotted) from the gel to a polyvinylidene difluoride (PVDF) or nitrocellulose membrane in electric current [98,99]. In the final step, the protein of interest immobilized on the membrane is visualized, using a specific labeled antibody as a detection probe [100,101]. Viral proteins are usually detected by a combination of antibodies—primary protein-specific antibody and secondary antibody labeled by a fluorescent dye or an enzyme [102].

#### 3.1.2. Detection of Viral Nucleic Acids

Nowadays, molecular biology techniques make it possible to detect specific nucleic acid sequences of pathogens, including viruses, in clinical and environmental samples. A variety of protocols may be employed to extract and purify nucleic acids by removing cell debris and inhibitors [103]. After nucleic acid extraction, DNA or RNA can be analyzed using hybridization techniques, namely Southern blot, Northern blot, or dot blot. In hybridization assays, a labeled oligonucleotide probe that is complementary to the genomic sequence of interest is used to demonstrate the presence of a particular sequence in the sample. However, these traditional molecular methods have disadvantages of being labor-intensive, cumbersome, and requiring large amounts of nucleic acids, so they are not routinely used in diagnostic laboratories [104,105].

Significant improvements in detection sensitivity over direct hybridization have been achieved by nucleic acid amplification methods such as polymerase chain reaction (PCR), reverse transcription PCR (RT-PCR) or quantitative real-time PCR (qPCR). Other benefits of PCR-based methods are high reproducibility, rapidity, and cost-effectiveness [106]. However, the presence of inhibitory substances, that are often co-concentrated with viruses, still represent a limitation in the PCR analysis of environmental water samples. Additionally, PCR-based methods are not appropriate for differentiating between infective and non-infective viruses [87,107].

PCR is one of the most widely used methods for the detection of viruses in environmental samples. During PCR, a specific target sequence from a complex pool of DNA is amplified in a three-step thermal cycling process—denaturation (94 °C), annealing (40 °C–65 °C), and extension (72 °C) [108,109]. The process requires the use of specific forward and reverse primers—short synthetic oligonucleotides designed to be complementary to target sites on the template DNA. The reaction mixture also includes thermostable DNA polymerase and four deoxyribonucleoside triphosphates (dNTPs) in a buffer solution. After 20–40 PCR cycles, amplicons of the expected size may be detected by agarose gel electrophoresis and staining with ethidium bromide [110]. Moreover, the identity of the amplified PCR products can be confirmed by sequencing or by hybridization with internal nucleotide probes [109].

Modifications of the basic PCR protocol have been introduced to increase sensitivity, specificity, and efficiency of virus detection. Nested PCR involves the use of two primer sets (outer pair and inner pair) in two successive PCR runs. The inner primers are designed to bind the area within the sequence amplified in the first PCR run, so that the initial PCR product can be used as the template for the second PCR run. If one of the primers in the second set is the same as for the first amplification, then the method is called semi-nested PCR [103,111]. Multiplex PCR is a variation of conventional PCR that allows the simultaneous detection of several viruses through the employment of multiple primer sets within a single PCR mixture to amplify sequences of varying sizes that are specific for each virus targeted [109,112].

PCR cannot be used to amplify RNA molecules directly. Therefore, PCR-based methods require an extra reverse transcription (RT) step in order to identify RNA viruses. RT can be initiated by oligo(dT) primers, random oligonucleotide primers, or sequence-specific primers. The reaction is catalyzed by enzyme reverse transcriptase, which can transcribe an RNA template into a complementary DNA (cDNA). Thereafter, the cDNA can serve as a template for PCR amplification. RT-PCR can be generally performed either in a one-step (RT carried out in the same tube as PCR) or two-step (RT carried out in a separate reaction) format [113,114].

qPCR enables the monitoring of DNA amplification in real time by measuring fluorescent signals; thus, the need for post-amplification confirmative analyses is eliminated. There are two strategies for the real-time detection of amplified PCR products—non-specific binding of fluorescent intercalating dye (SYBR Green I, EvaGreen) to double-stranded DNA, and sequence-specific hybridization of fluorescently labeled oligonucleotide probes (TaqMan probes, molecular beacons). In qPCR, fluorescence is measured after each cycle, and the intensity of the fluorescent signal reflects the momentary amount of DNA amplicons in the sample at that specific time [115,116]. The appropriate calibration of qPCR assays and the use of standard curves allows the assessment of absolute copy numbers of the target of interest. qPCR is highly sensitive—the limit of detection for a well-optimized qPCR assay can reach as low as 1 to 10 target molecules per reaction [117]. For analysis of relative gene expression, both RT-PCR and qPCR are merged, and this combined method is termed quantitative RT-PCR (qRT-PCR) [118]. The relative expression of a target gene is measured in relation to reference gene(s) as endogenous control(s). qRT-PCR can be performed in a one-step or a two-step assay [119]. Multiplexing in the qPCR assay is also possible, but it requires the use of probes with different kinds of fluorophores [120].

In recent years, digital PCR (dPCR) has gained attention as a novel approach to detect and quantify nucleic acids. The major benefit of dPCR over qPCR is the direct absolute quantification of virus genome copy numbers in a sample without the necessity of external calibration. This improvement is achieved by partitioning of amplification reactions into thousands of small reaction volumes (typically nanoliters) so that each individual reaction mixture contains zero or a single copy of the target molecule [87,114]. After thermal cycling and read-out, each miniature reaction can be scored as either positive (fluorescent) or negative; thus, the Poisson statistics can be performed for calculation of the initial number of targets [121]. dPCR platforms can generally be divided into two groups: droplet dPCR (emulsion based) and chip-based dPCR (microfluidic). Unlike qPCR, dPCR has also the advantage of being more tolerant to some PCR inhibitors. Moreover, the results obtained with dPCR are very precise and accurate, even at very low target copy numbers [122].

The current situation brings with it various new or innovative techniques for the detection of SARS-CoV-2 proteins in wastewater. One of these methods is, for example, utilization of the MPAD technique. MPAD offers easier SARS-CoV-2 protein detection in wastewater compared to the PCR technique [123]. Another method is LAMP, in the form RT-qLAMP, namely loop-mediated isothermal amplification, which is suitable for detection of SARS-CoV-2 virus in very low concentrations due to the low reported cases rates of r.g. 1–10 per 100,000 people. The LAMP technique therefore seems to be more appropriate than the PCR method because it is less time consuming [124].

#### 3.1.3. Latest Research in Traditional Techniques for Virus Detection

Lately, sequencing of the wastewater or sludge was introduced to wastewater monitoring for microbial and viral presence [125]. The technique is based on reading signals of single nucleotides one by one in the order of all the fragments of nucleic acids present in the sample and collecting large datasets that are afterwards analyzed, assembled through bioinformatic tools, and compared to an open-source database with the reference genome [126].

Studies differentiating bacterial taxa were based on the sequencing of 16S fragments of bacterial ribosomal DNA and their presence in wastewater. 16s rDNA sequencing is based on partial similarity in the complete gene with an approximate length of 1540 bases and variety in nine hypervariable regions unique for each bacterial genus [127]. Unfortunately, the first and second generation of sequencing depend on a relatively large and stationary apparatus with high running costs [128]. This changed after the introduction of the third generation of sequencers developed by Oxford Nanopore Technology. Their product, MinION, is a USB-shaped sequencing device connected to a standard computer that can be applied also in the field [129].

With the evolving situation of the SARS-CoV-2 pandemic and the proven presence of virus in human feces and subsequent confirmation in wastewater, sequencing technology again has gained its importance [28]. Whole genome sequencing of the virus in wastewater can reveal possible mutations occurring in population that are going to be more frequent after the introduction of broad vaccination and convalescent plasma treatment [130]. Furthermore, the results of such an analysis are in advance of clinical testing and sequencing. This happens because shedding of the virus into the feces occurs moments after infection, while it takes time for the infected individual to evolve symptoms and undergo clinical testing. Several studies showed that sequencing of SARS-CoV-2 in wastewater revealed genotypes already circulating in communities as well as novel metagenomic variants not yet detected by clinical testing [131,132,133].

The importance of the involvement of the technique for regular testing will rely on the creation of standardized protocols for sampling, pre-treatment, concentration, and extraction of the sample, including sequencing [134]. Nowadays, many researchers are looking for optimal solutions for given challenges. After establishing standardized protocols, it will be possible to analyze microbial as well as viral presence in the wastewater as is already done in the case of drugs, pesticides, and other harmful agents.

### 3.2. Biosensors—Early Warning System for Virus Detection in Wastewater

Since the outbreak of COVID-19, RT-PCR methods have been routinely used to detect SARS-CoV-2 in many research and disease control centers. However, they have some disadvantages that limit on-site and real-time monitoring of samples, such as expensive equipment, necessary qualified technicians, complicated handling of samples in the laboratory, and the long time required for data processing and analysis (up to 4–6 h). For this reason, it is necessary to develop analytical equipment for fast and accurate detection of viruses at wastewater collection points without the need for centralized laboratories [135].

For example, Mene et al. presented a multifunctional fluorescent protein nanowire containing green fluorescent protein molecules for the detection of hemagglutinin 1 from influenza virus together with proteins p24 and gp120 from HIV [136].

Yang et al. developed an electrochemical biosensor for DNA extraction from wastewater, which uses synthesized ferrocenyl incorporated into double-stranded DNA, and that serves as a redox marker. A few years ago, a highly sensitive graphene-based electrochemical biosensor for rotavirus detection was also invented. The reduced graphene oxide film significantly improved the sensor’s ability to capture viral cells through antibody–antigen interactions [137].

Paper analytical devices have also been developed to filter pathogenic genetic material from wastewater samples by biochemical reaction. These devices can detect if SARS-CoV-2 genetic material is present in the sample. The cost is relatively low (less than USD 1.25) and the detection time is about 30 min. The results are visible to the naked eye, with a green circle indicating positive and a blue circle indicating negative. Although these sensors are fast and easy to use, they tend to have lower sensitivity, accuracy, and specificity than PCR methods. It is also important to note that water is a complex matrix and contains various interfering components that need to be removed (e.g., by purifying the sample) to enhance the accuracy and practicality of paper biosensors. However, fast detection, ease of use, and portability are the main advantages of biosensors, making them suitable for use in detecting viruses in wastewater samples [138].

Traditional diagnosis and laboratory techniques are usually not suitable for fast on-site analysis, as they require virus isolation and biocontainment, the ability to grow cultured cells for cytopathology related assays, and/or usually expensive laboratory equipment that are difficult to transport and use at the point of care. Other disadvantages of commonly used laboratory-based procedures that increase time-to-answer and costs are time demands, the requirement for samples to be transported to centralized diagnostic laboratories, labor-intensiveness, the requirement of highly qualified operators, and sometimes lower sensitivity at relatively higher detection limits [139,140].

(Bio)sensors are able to analyze various types of substances. Viruses belong to the large group of analytes that can be detected and quantified by several types of (bio)sensors. Viral biosensors offer excellent alternatives to conventional diagnostic methods and have great potential to provide selective, sensitive, low-cost, rapid, and portable devices.

Generally, sensors are devices that are composed of two basic parts: (i) (bio)chemical or biological recognition layers (receptors) that are responsible for the interaction with a target analyte, and (ii) a transducer that converts the recognition process into a signal (electrochemical, optical, calorimetric, acoustic) that can be further processed and quantified [141].

They are highly sensitive and selective, with simple operation and reparation, portability, and fast analysis [142]. Electrochemical (bio)sensors are based on reaction with the (bio)chemical environment and produce a proportional electrical signal to the target molecule amount [143]. The electrochemical techniques utilized to measure the analyte concentrations can be divided into several groups: potentiometric, voltammetric, conductometric, amperometric, and impedimetric techniques [144].

The aim of this part of the review is to give a brief description of recent developments in electrochemical biosensors for viruses and viral infections. The text divides biosensors according to the recognition layer to: (i) biocatalytic sensors and (ii) affinity sensors. It must be noted that this short contribution is intentionally not all inclusive given the large numbers of publications in this field.

#### 3.2.1. Biocatalytic Sensors

These types of sensors use immobilized enzymes, cells, and whole tissues as a recognition layer. Enzyme-based biocatalytic biosensors utilize the catalytic properties of enzymes. Therefore, they are very selective, sensitive, and very effective. Enzymes can be immobilized by physical interactions (e.g., adsorption) and covalently bound either directly to the electrode surface, nanoparticles, or some interlayer or mixed with electrode material (paste electrodes).

Ilkhani et al. constructed an impedimetric biosensor for the detection of Ebola virus DNA by enzyme-amplified detection using streptavidin–alkaline phosphatase conjugate [145,146]. The detection limit was 4.7 nM using this biosensor. The authors also confirmed high selectivity and reproducibility of this impedimetric biosensor. Another example of an enzyme-based biosensor is a disposable microfluidic electrochemical device for citrus tristeza virus (Closteroviridae; a filamentous positive strand RNA virus) [147,148]. The detection is based on the enzymatic activity of horseradish peroxidase (HRP) using H_2_O_2_ and hydroquinone, and the detection limit was 0.3 fg/mL. Oxidation of 3,3,5,5-tetramethylbenzidine substrate by HRP enzyme for the simultaneous detection of three influenza virus strains (H1N1, H5N1, and H7N9) was used in the work of Han et al. [149]. In this work, amperometry was applied to detect H1N1, H5N1, and H7N9 influenza viruses simultaneously in a mixture of three virus antigens using three-gold electrodes and ZnO nanorods for sensitivity enhancement. The limit of detection of each virus using this technique was 1 pg/mL [148,149].

#### 3.2.2. Affinity Biosensors

Very important parts of the recognition layer of these biosensors are usually aptamers, nucleic acids, membrane receptors, and antibodies. These biological components can be directly bound to the electrode surface, connected with a working electrode via some interlayer(s) (human/bovine serum albumin) and/or are bound to nanoparticles.

A huge group of biosensors is based on the various types of nanoparticles [150]. Electrochemical impedance spectroscopy (EIS, impedimetric biosensors) was used for the determination of influenza M1 protein on nanocrystalline boron doped diamond in saliva [151]; influenza A virus on graphene–gold hybrid nanoparticles, where the detection was based on the neuraminidase activity [152]; human papilloma virus DNA, using golden nanotubes based on nanoporous polycarbonate in electrical alignment [153] with LOD 1 fM; Zika virus-specific antibodies in infected individuals [154]; and dengue virus 2 NS1 antibody [155] based on carboxylated and non-carboxylated CNTs, respectively. EIS has also been used for Japanese encephalitis virus in human serum, where carbon nanoparticles were used and the LOD was down to 0.36 ng/mL [156].

Various types of voltammetric techniques in combination with nanostructured supported material were used to determine avian influenza virus (H5N1) in chicken serum on porous Au nanoparticles [157] with an LOD of 1 pM, Hepatitis B e antigen detection based on a signal amplification by co-catalysis of horseradish peroxidase and nanoporous gold with an LOD of 64 fg/mL in human serum [158], human enterovirus 71 with dual-labeled magnetic nanobeads [159] with an LOD of 10 pg/mL, and many others [150]. The electrochemical immunosensor for corona virus associated with the Middle East respiratory syndrome (MERS) using an array of gold nanoparticle-modified carbon electrodes with voltammetric detection has been already designed [160]. The voltammetric response is detected by monitoring the change in the peak current after addition of different concentrations of antigen against MERS-CoV. The test was finished in 20 min with detection limits of 400 fg and 1000 pg/mL for HCoV and MERS-CoV, respectively.

In clinical samples, a photoelectrochemical immunosensor based on gold nanoparticle/ZnAgInS quaternary quantum dots was successfully used for the high-performance determination of hepatitis B virus surface antigen with an LOD of 500 fg/mL.

Chronoamperometric detection was, for example, utilized to determine influenza virus H9N2 based on both immunomagnetic extraction and gold catalysis using an immobilization-free screen-printed carbon microelectrode [161]. This approach allows for the rapid detection of influenza virus A (H9N2) at a less than 16 hemagglutination unit (HAU) titer. Another example of an amperometric biosensor type is a device based on Au@Pd nanoparticles loaded by molybdenum disulfide (MoS2) functionalized multiwalled carbon nanotubes (MWCNTs) that has, according to the authors, better electrocatalytic activity towards reduction of H_2_O_2_ for the detection of hepatitis B e antigen [162], and a biosensor based on magnetic bead/capture DNA/glucose-loaded nanoliposomes for the rapid and direct screening of hepatitis C virus RNA with an LOD of 1.9 pM in human serum [163].

Nucleic acid-based biosensors compose another important group of devices for viral detection [164,165,166]. In the case of a DNA biosensor, single stranded DNA is immobilized on an electrode (nanoparticle) surface to detect its complementary DNA/RNA sequence due to surface hybridization. A label-free electrochemical DNA biosensor was used for Zika virus determination using disposable electrodes in one sample drop with an LOD of 25 nM [167]. An ultrasensitive impedimetric biosensor was developed for the determination of human papilloma virus DNA on nanoporous polycarbonate in electrical alignment. The EIS response was intensified by AuNTs and an electric field to acquire an LOD of 1 fM [153]. A thiolated DNA probe immobilized and optimized for DNA hybridization detection was utilized for positive-sense single-stranded RNA Citrus tristeza virus, showing a logarithmic relation from 100 nM to 10 μM [168].

An interesting group of sensors for virus detection are molecularly imprinted (MI) biosensors [169]. A molecularly imprinted electrochemiluminescence sensor using EuS nanocrystals as the luminophore was used for ultrasensitive HIV-1 gene detection. This sensor was evaluated for analysis of the HIV-1 gene in real human serum at a range of 3.0 fM to 0.3 nM and an LOD of 300 aM [170]. MI biosensors were also successfully used for the detection of influenza virus (H5N1, H5N3, H1N1, H1N3, H6N1) [171,172], Dengue virus [173], adenovirus [174], picornavirus [175], and others [169].

Almost all biosensors are focused on the determination of viruses in clinical samples such as serum, blood, saliva, tears, and urine. However, with the outbreak of COVID 19 caused by the SARS-CoV-2 virus, and with the knowledge that this virus can be excreted via feces and urine and, therefore, goes to the sewage system [176], one can ask if all/some of these devices can be easily switched to also determine viruses in wastewaters such as other micropollutants. Paper-based biosensors have been proven to be effective for infectious diseases and virus detection and can be a very simple, portable, and inexpensive alternative to conventional techniques, e.g., RT PCR in the case of SARS-CoV-2 [177]. One example of a paper device made from cellulosic paper and a flexible plastic plate is the electrochemical sensor for HIV detection. Other types of viruses, such as rotavirus A, Zika virus, and human papillomavirus have also been detected by paper-type devices with different detection methods [177,178,179].

**Figure 2:**
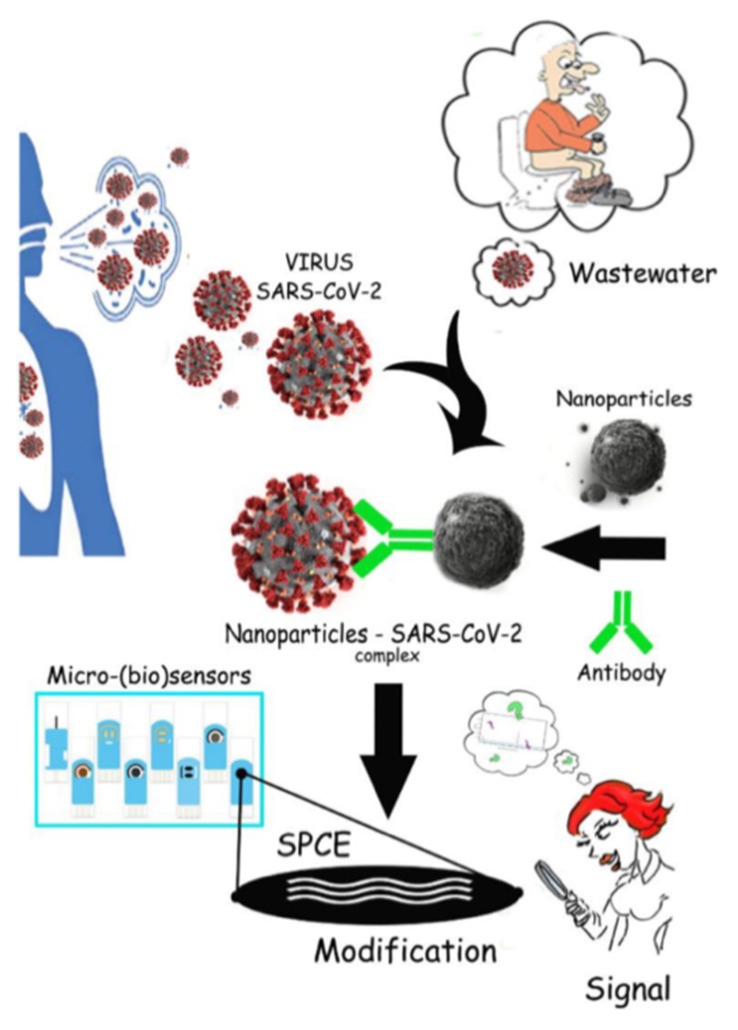
The usage of biosensors in sewerage system.

The installation of automatic samplers or specific types of microsensors in the sewer system, which could react with sufficient sensitivity not only to selected SARS-CoV-2 virus fragments or biomarkers in the future, might gradually lead to the creation of a SMART system that could identify a COVID-19 outbreak in a city faster than established epidemiological procedures (Figure 2).

## 4. Conclusions

At present, scientific teams from several countries around the world, whether at the national or transnational level, are engaged in wastewater monitoring to determine drug consumption in the region. Research in this area has been carried out for about fifteen years, resulting in the development of new analytical procedures, the possibility of monitoring new types of drugs, or the quantitative measurement of specific biomarkers in wastewater from different regions and cities, for example enabling us to assess the lifestyle of the population. Therefore, the serious question arises whether, in the current COVID-19 pandemic induced by the SARS-CoV-2 coronavirus, all of the acquired knowledge and experience can be used to identify outbreaks of a certain type of virus. At the moment, we do not know. Several assumptions and ideas have been made but these need to be confirmed by studies to ensure that wastewater monitoring for early detection of possible outbreaks of COVID-19-like diseases in the population is a validated and recognized detection methodology in broad scientific circles. Conventional laboratory techniques are, however, usually not suitable for rapid on-site analyses, because they are time demanding, require samples to be transported to centralized diagnostic labs and highly educated operators, etc. Electrochemical techniques in combination with proper biosensors are suitable for direct, real time, and on-site viral detection. Biosensors can be distributed at various points in the sewage system to create an alert system for an impending epidemic. This can also be digitized as an interactive map. Miniaturization allows for the preparation of sensor chips for the simultaneous monitoring of various types of viruses depending on the microelectrode modifications.

Early detection of outbreaks can thus be recorded mainly on the basis of a change in its occurrence over time in combination with parameters characterizing the surveyed population and consistently defined sampling points in the sewerage network.

## Figures and Tables

**Table 1:** Overview of some important wastewater-based epidemiology studies for SARS-CoV-2.

SARS-CoV-2 Gene	Source	Date of Sample Collection	Ref.
*N1, N2, N3, E*	Netherlands	5 February–25 March 2020	[21]
*E, N2*	Helsinki, Finland	19–20 April and 24–25 May 2020	[23]
*N1, N2, N3*	Region of Murcia, Spain	12 March–14 April 2020	[31]
*ORF1a, S, N1, N2*	Yamanashi Prefecture, Japan	17 March–7 May 2020	[32]
*RdRp, E, N, M*	Germany	8–9 May 2020	[41]
*N1, N2, N3*	Massachusetts, USA	18–25 March 2020	[48]
*N*	Brisbane, Australia	27 March–1 April 2020	[49]
*RdRp, E*	Paris, France	5 March–23 April 2020	[50]
*ORF1ab, E, N*	Milan and Monza, Italy	14 and 22 April 2020	[52]
*RdRp*	Istanbul, Turkey	7 May 2020	[55]
*ORF1ab, N*	Bangladesh	10 July–29 August 2020	[71]
*ORF1ab, N, S*	Dubai, UAE	22 and 28 April 2020	
		4 May 2020	[72]
		7 May–7 July 2020	
*ORF1ab, N, S*	Ahmedabad, India	8 and 27 May 2020	[73]
*ORF1ab, S, RdRp*	Milan and Rome, Italy	3 February–2 April	[74]
*E*	Israel	10 March–21 April 2020	[75]
*N1, N2*	Quito, Ecuador	5 June 2020	[76]

## Data Availability

Not applicable.

## References

[1] Daughton C.D., Ternes T.A. (1999). Pharmaceuticals and personal care products in the environment: Agents of subtle change?. Environ. Health Perspect..

[2] Zuccato E., Chiabrando C., Castiglioni S., Calamari D., Bagnati R., Schiarea S., Fanelli R. (2005). Cocaine in surface waters: A new evidence-based tool to monitor community drug abuse. Environ. Health.

[3] Bijlsma L., Celma A., Lopez F.J., Hernandez F. (2019). Monitoring new psychoactive substances use through wastewater analysis: Current situation, challenges and limitations. Curr. Opin. Environ. Sci. Health.

[4] Castrignanò E., Yang Z., Feil E.J., Bade R., Castiglioni S., Causanilles A., Gracia-Lor E., Hernandez F., Plosz B.G., Ramin P. (2020). Enantiomeric profiling of quinolones and quinolones resistance gene qnrS in European wastewaters. Water Res..

[5] Gracia-Lor E., Zuccato E., Hernandez F., Castiglioni S. (2020). Wastewater-based epidemiology for tracking human exposure to mycotoxins. J. Hazard. Mater..

[6] Rousis I.N., Gracia-Lor E., Reid M.J., Baz-Lomba J.A., Ryu Y., Zuccato E., Thomas K.V., Castiglioni S. (2020). Assessment of human exposure to selected pesticides in Norway by wastewater analysis. Sci. Total Environ..

[7] Sims N., Kasprzyk-Hordern B. (2020). Future perspectives of wastewater-based epidemiology: Monitoring infectious disease spread and resistance to the community level. Environ. Int..

[8] Castrignanò E., Mardal M., Rydevik A., Miserez B., Ramsey J., Shine T., Pantos D., Meyer M.R., Kasprzyk-Hordern B. (2017). A newapproach towards biomarker selection in estimation of human exposure to chiral chemicals: A case study of mephedrone. Sci. Rep..

[9] Xagoraraki I., O’Brien E., O’Bannon D. (2020). Wastewater-based epidemiology for early detection of viral outbreaks. Women in Water Quality, Women in Engineering and Science.

[10] (2020). WHO. https://web.archive.org/web/20141214011751/.

[11] (2020). WHO. https://www.who.int/en/news-room/fact-sheets/detail/rabies.

[12] (2020). WHO. https://www.who.int/csr/sars/country/table2004_04_21/en/.

[13] Ozili P.K., Arun T. (2020). Spillover of COVID-19: Impact on the global economy. SSRN Electron. J..

[14] Van Doremalen N., Bushmaker T., Morris D.H., Holbrook M.G., Gamble A., Williamson B.N., Tamin A., Harcourt J.L., Thornburg N.J., Gerber S.I. (2020). Aerosol and surface stability of SARS-CoV-2 as compared with SARS-CoV-1. N. Engl. J. Med..

[15] Tian Y., Rong L., Nian W., He Y. (2020). Review article: Gastrointestinal features in COVID-19 and the possibility of faecal transmission. Aliment. Pharmacol. Ther..

[16] Wang D., Hu B., Hu C., Zhu F., Liu X., Zhang J., Wang B., Xiang H., Cheng Z., Xiong Y. (2020). Clinical characteristics of 138 hospitalized patients with 2019 novel coronavirus-infected pneumonia in Wuhan, China. J. Am. Med. Assoc..

[17] WHO (2020). Water, Sanitation, Hygiene and Waste Management for COVID-19. https://www.who.int/publications-detail/water-sanitation-hygiene-and-waste-management-for-covid-19.

[18] Xiao E., Tang M., Zheng X., Liu Y., Li C., He J., Hong Z., Huang S., Zhang Z., Lin X. (2020). Evidence for gastrointestinal infection of SARS-CoV. Gastroenterology.

[19] Yeo C., Kaushal S., Yeo D. (2020). Enteric involvement of coronaviruses: Is faecal–oral transmission of SARS-CoV-2 possible?. Lancet Gastroenterol. Hepatol..

[20] Langone M., Petta L., Cellamare C.M., Ferraris M., Guzzinati R., Mattioli D., Sabia G. (2020). SARS-CoV-2 in water services: Presence and impacts. Environ. Pollut..

[21] Medema G., Heijnen L., Elsinga G., Italiaander R., Brouwer A. (2020). Presence of SARS-Coronavirus-2 RNA in Sewage and Correlation with Reported COVID-19 Prevalence in the Early Stage of the Epidemic in The Netherlands. Environ. Sci. Technol. Lett..

[22] Orive G., Lertxundi U., Barcelo D. (2020). Early SARS-CoV-2 outbreak detection by sewage-based epidemiology. Sci. Total Environ..

[23] Hokajärvi A.M., Rytkönen A., Tiwari A., Kauppinen A., Oikarinen S., Lehto K.M., Kankaanpää A., Gunnar T., Al-Hello H., Blomqvist S. (2021). The detection and stability of the SARS-CoV-2 RNA biomarkers in wastewater influent in Helsinki, Finland. Sci. Total. Environ..

[24] Pan Y., Zhang D., Yang P., Poon L.L.M., Wang Q. (2020). Viral load of SARS-CoV-2 in clinical samples. Lancet Infect. Dis..

[25] Woelfel R., Corman V.M., Guggemos W., Seilmaier M., Zange S., Mueller M.A., Niemeyer D., Vollmar P., Rothe C., Hoelscher M. (2020). Clinical presentation and virological assessment of hospitalized cases of coronavirus disease 2019 in a travel-associated transmission cluster. medRxiv.

[26] Chen C., Gao G., Xu Y., Pu L., Wang Q., Wang L., Wang W., Song Y., Chen M., Wang L. (2020). SARS-CoV-2–positive sputum and feces after conversion of pharyngeal samples in patients with COVID-19. Ann. Intern. Med..

[27] Lescure F.X., Bouadma L., Nguyen D., Parisey M., Wicky P.H., Behillil S., Gaymard A., Bouscambert-Duchamp M., Donati F., Le Hingrat Q. (2020). Clinical and virological data of the first cases of COVID-19 in Europe: A case series. Lancet Infect. Dis..

[28] Wang W., Xu Y., Gao R., Lu R., Han K., Wu G., Tan W. (2020). Detection of SARS-CoV-2 in different types of clinical specimens. J. Am. Med. Assoc..

[29] Wu Y., Guo C., Tang L., Hong Z., Zhou J., Dong X., Yin H., Xiao Q., Tang Y., Qu X. (2020). Prolonged presence of SARS-CoV-2 viral RNA in faecal samples. Lancet Gastroenterol. Hepatol..

[30] Xu Y., Li X., Zhu B., Liang H., Fang C., Gong Y., Guo Q., Sun X., Zhao D., Shen J. (2020). Characteristics of pediatric SARS-CoV-2 infection and potential evidence for persistent fecal viral shedding. Nat. Med..

[31] Randazzo W., Truchado P., Cuevas-Ferrando E., Simon P., Allende A., Sanchez G. (2020). SARS-CoV-2 RNA in wastewater anticipated COVID-19 occurrence in a low prevalence area. Water Res..

[32] Haramoto E., Malla B., Thakali O., Kitajima M. (2020). First environmental surveillance for the presence of SARS-CoV-2 RNA in wastewater and river water in Japan. Sci. Total Environ..

[33] Randazzo W., Cuevas-Ferrando E., Sanjuan R., Domingo-Calap P., Sanchez G. (2020). Metropolitan wastewater analysis for COVID-19 epidemiological surveillance. Int. J. Hyg. Environ. Health..

[34] Fongaro G., Stoco P.H., Souza D.S.M., Grisard E.C., Magri M.E., Rogovski P., Schorner M.A., Barazzetti F.H., Christoff A.P., de Oliveira L.F.V. (2021). SARS-CoV-2 in human sewage in Santa Catalina, Brazil, November 2019. Sci. Total Environ..

[35] La Rosa G., Mancini P., Ferraro G.B., Veneri C., Iaconelli M., Bonadonna L., Lucentini L., Suffredini E. (2021). SARS-CoV-2 has been circulating in northern Italy since December 2019: Evidence from environmental monitoring. Sci. Total Environ..

[36] Li R., Pei S., Chen B., Song Y., Zhang T., Yang W., Shaman J. (2020). Substantial undocumented infection facilitates the rapid dissemination of novel coronavirus (SARS-CoV-2). Science.

[37] Kitajima M., Ahmed W., Bibby K., Carducci A., Gerba C.P., Hamilton K.A., Harmoto E., Rose J.B. (2020). SARS-CoV-2 in wastewater: State of the knowledge and research needs. Sci. Total Environ..

[38] La Rosa G., Bonadonna L., Lucentini L., Kenmore S., Suffredini E. (2020). Coronavirus in water environments: Occurrence, persistence and concentration methods—A scoping review. Water Res..

[39] Bivins A., Greaves J., Fischer R., Yinda K.C., Ahmed W., Kitajima M., Munster V.J., Bibby K. (2020). Persistence of SARS-CoV-2 in Water and Wastewater. Environ. Sci. Technol. Lett..

[40] Jones D.L., Baluja M.Q., Graham D.W., Corbishley A., McDonald J.E., Malham S.K., Hillary L.S., Connor T.R., Gaze W.H., Moura I.B. (2020). Fecal Shedding of SARS-CoV-2 and its Potential Role in Person-To-Person Transmission and the Environment- Based Spread of COVID-19. Sci. Total Environ..

[41] Westhouse S., Weber F.A., Schiwy S., Linnemann V., Brinkmann M., Widera M., Greve C., Janke A., Hollert H., Wintgens T. (2021). Detection of SARS-CoV-2 in raw and treated wastewater in Germany—Suitability for COVID-19 surveillance and potential transmission risks. Sci. Total Environ..

[42] Hart O.E., Halden R.U. (2020). Computational analysis of SARS-CoV-2/COVID-19 surveillance by wastewater-based epidemiology locally and globally: Feasibility, economy, opportunities and challenges. Sci. Total Environ..

[43] Katayama H., Haramoto E., Oguma K., Yamashita H., Tajima A., Nakajima H., Ohgaki S. (2008). One-year monthly quantitative survey of noroviruses, enteroviruses, and adenoviruses in wastewater collected from six plants in Japan. Water Res..

[44] Skraber S., Ogorzaly L., Helmi K., Maul A., Hoffmann L., Cauchie H.M., Gantzer C. (2009). Occurrence and persistence of enteroviruses, noroviruses and F-specific RNA phages in natural wastewater biofilms. Water Res..

[45] Bashir M.F., Ma B., Bilal, Komal B., Bashir M.A., Tan D., Bashir M. (2020). Correlation between climate indicators and COVID-19 pandemic in New York, USA. Sci. Total Environ..

[46] Gupta S., Parker J., Smits S., Underwood J., Dolwani S. (2020). Persistent viral shedding of SARS-CoV-2 in faeces—A rapid review. Colorectal Dis..

[47] Núñez-Delgado A. (2020). What do we know about the SARS-CoV-2 coronavirus in the environment?. Sci. Total Environ..

[48] Wu F.Q., Xiao A., Zhang J.B., Gu X.Q., Lee W.L., Kauffman K., Hanage W.P., Matus M., Ghaeli N., Endo N. (2020). SARS-CoV-2 titers in wastewater are higher than expected from clinically confirmed cases. mSystems.

[49] Ahmed W., Angel N., Edson J., Bibby K., Bivins A., O’Brien J.W., Choi P.M., Kitajima M., Simpson S.L., Li J. (2020). First confirmed detection of SARS-CoV-2 in untreated wastewater in Australia: A proof of concept for the wastewater surveillance of COVID-19 in the community. Sci. Total Environ..

[50] Wurtzer S., Marechal V., Mouchel J.M., Moulin L. (2020). Time course quantitative detection of SARS-CoV-2 in Parisian wastewaters correlates with COVID-19 confirmed cases. medRxiv.

[51] Kocamemi B.A., Kurt H., Hacioglu S., Yarali C., Saatci A.M., Pakdemirli B. (2020). First Data-Set on SARS-CoV-2 Detection for Istanbul Wastewaters in Turkey. medRxiv.

[52] Rimoldi S.G., Stefani F., Gigantiello A., Polesello S., Comandatore F., Mileto D., Maresca M., Longobardi C., Mancon A., Romeri F. (2020). Presence and infectivity of SARS-CoV-2 virus in wastewaters and rivers. Sci. Total Environ..

[53] Iglesias N.G., Gebhard L.G., Carballeda J.M., Aiello I., Recalde E., Terny G., Ambrosolio S., L’Arco G., Jonatan K., Brardinelli J.I. (2020). SARS-CoV-2 surveillance in untreated wastewater: First detection in a low-resource community in Buenos Aires, Argentina. medRxiv.

[54] Sherchan S.P., Shahin S., Ward L.M., Tandukar S., Aw T.G., Schmitz B., Ahmed W., Kitajima M. (2020). First detection of SARS-CoV-2 RNA in wastewater in North America: A study in Louisiana, USA. Sci. Total Environ..

[55] Kocamemi B.A., Kurt H., Sait A., Sarac F., Saatci A.M., Pakdemirli B. (2020). SARS-CoV-2 Detection in Istanbul Wastewater Treatment Plant Sludges. medRxiv.

[56] Miyani B., Fonoll X., Norton J., Mehrotra A., Xagoraraki I. (2020). SARS-CoV-2 in Detroit Wastewater. J. Environ. Eng..

[57] Mlejnková H., Sovova K., Vasickova P., Ocenaskova V., Jasikova L., Juranova E. (2020). Preliminary Study of Sars-Cov-2 Occurrence in Wastewater in the Czech Republic. Int. J. Environ. Res. Public Health.

[58] Agrawal S., Orschler L., Lackner S. (2021). Long-term monitoring of SARS-CoV-2 in wastewater of the Frankfurt metropolitan area in Southern Germany. Sci. Rep..

[59] Been F., Rossi L., Ort C., Rudaz S., Delemont O., Esseiva P. (2014). Population normalization with ammonium in wastewater-based epidemiology: Application to illicit drug monitoring. Environ. Sci. Technol..

[60] Chen C., Kostakis C., Gerber J.P., Tscharke B.J., Irvine R.J., White J.M. (2014). Towards finding apopulation biomarker for wastewater epidemiology studies. Sci. Total Environ..

[61] Choi M.P., Tscharke B.J., Donner E., O’Brien J.W., Grant S.C., Kaserzon S.L., Mackie R., O’Malley E., Crosbie N.D., Thomas K.V. (2018). Wastewater-based epidemiology biomarkers: Past, present and future. Trends Anal. Chem..

[62] Lomba B.A.J., Di Ruscio F., Amador A., Reid M., Thomas K.V. (2019). Assessing alternative population size proxies in a wastewater catchment area using mobile device data. Environ. Sci. Technol..

[63] Larson R.C., Berman O., Nourinejad M. (2020). Sampling manholes to home in on SARS-CoV-2 infections. PLoS ONE.

[64] Daughton C. (2020). The international imperative to rapidly and inexpensively monitor community-wide Covid-19 infection status and trends. Sci. Total Environ..

[65] Farkas K., Walker D.I., Adriaenssens E.M., McDonald J.E., Hillary L.S., Malham S.K., Jones D.L. (2020). Viral indicators for tracking domestic wastewater contamination in the aquatic environment. Water Res..

[66] Thomas K.V., Bijilsma L., Castiglioni S., Covaci A., Emke E., Grabic R., Hernandez F., Karolak S., Kasprzyk-Hordern B., Lindeberg R.H. (2012). Comparing illicit drug use in 19 European cities through sewage analysis. Sci. Total Environ..

[67] Ort C., van Nuijs A.L.N., Berset J.D., Bijlsma L., Castiglioni S., Covaci A., de Voogt P., Emke E., Fatta-Kassinos D., Griffiths P. (2014). Spatial differences and temporal changes in illicit drugs use in Europe quantified by wastewater analysis. Addiction.

[68] Krizman-Matasic I., Senta I., Kostanjevecki P., Ahel M., Terzic S. (2019). Long-term monitoring of drug consumption patterns in a large-sized European city using wastewater-based epidemiology: Comparison of two sampling schemes for the assessment of multiannual trends. Sci. Total Environ..

[69] COVID19 Wbe Collaborative. https://www.covid19wbec.org/.

[70] Polo D., Quintela-Baluja M., Corbishley A., Jones D.L., Singer A.C., Graham D.W., Romalde J.L. (2020). Making waves: Wastewater-based epidemiology for COVID-19—Approaches and challenges for surveillance and prediction. Water Res..

[71] Ahmed F., Islam A., Kumar M., Hossain M., Bhattacharya P., Islam T., Hossen F., Hossain S., Islam S., Uddin M. (2020). First detection of SARS-CoV-2 genetic material in the vicinity of COVID-19 isolation centre through wastewater surveillance in Bangladesh. medRxiv.

[72] Albastaki A., Naji M., Lootah R., Almeheiri R., Almulla H., Almarri I., Alreyami A., Aden A., Alghafri R. (2021). First confirmed detection of SARS-COV-2 in untreated municipal and aircraft wastewater in Dubai, UAE: The use of wastewater based epidemiology as an early warning tool to monitor the prevalence of COVID-19. Sci. Total Environ..

[73] Kumar M., Patel A.K., Shah A.V., Raval J., Rajpara N., Joshi M., Joshi C.G. (2020). First proof of the capability of wastewater surveillance for COVID-19 in India through detection of genetic material of SARS-CoV-2. Sci. Total Environ..

[74] La Rosa G., Iaconelli M., Mancini P., Bonanno F., Veneri C., Bonadonna L., Lucentini L., Suffredini E. (2020). First detection of SARS-CoV-2 in untreated wastewaters in Italy. Sci. Total Environ..

[75] Bar Or I., Yaniv K., Shagan M., Ozer E., Erster O., Mendelson E., Mannasse B., Shirazi R., Kramarsky-Winter E., Nir O. (2020). Regressing SARS-CoV-2 sewage measurements onto COVID-19 burden in the population: A proof-of-concept for quantitative environmental surveillance. medRxiv.

[76] Guerrero-Latorre L., Ballesteros I., Villacrés-Granda I., Grandam M.G., Freire-Paspuel B., Ríos Touma B. (2020). SARS-CoV-2 in river water: Implications in low sanitation countries. Sci. Total Environ..

[77] https://www.cdc.gov/coronavirus/2019-ncov/lab/rt-pcr-panel-primer-probes.html.

[78] Leland D.S., Ginocchio C.C. (2017). Role of cell culture for virus detection in the age of technology. Clin. Microbiol. Rev..

[79] Modrow S., Falke D., Truyen U., Schätzl H. (2013). Laboratory methods for detecting viral infections. Molecular Virology.

[80] Schauflinger M., Villinger C., Walther P., Bailer S.M., Lieber D. (2013). Three-dimensional visualization of virus-infected cells by serial sectioning: An electron microscopic study using resin embedded cells. Virus-Host Interactions: Methods and Protocols.

[81] Dilnessa T., Zeleke H. (2017). Cell Culture, Cytopathic effect and immunofluorescence diagnosis of viral infection. J. Microbiol. Modern. Tech..

[82] Stephenson J.R., Warnes A. (2011). Diagnostic Virology Protocols.

[83] Mattison K., Bidawid S. (2009). Analytical methods for food and environmental viruses. Food Environ. Virol..

[84] Barardi C.R.M., Viancelli A., Rigotto C., Correa A.A., Moresco V., Souza D.S.M., ElMahdy M.E.I., Fongaro G., Pilotto M.R., Nascimento M.A. (2012). Monitoring viruses in environmental samples. Int. J. Environ. Sci. Eng. Res..

[85] Calgua B., Barardi C.R.M., Bofill-Mas S., Rodriguez-Manzano J., Girones R. (2011). Detection and quantitation of infectious human adenoviruses and JC polyomaviruses in water by immunofluorescence assay. J. Virol. Methods.

[86] Greening G.E., Hewitt J., Lewis G.D. (2002). Evaluation of integrated cell culture-PCR (C-PCR) for virological analysis of environmental samples. J. Appl. Microbiol..

[87] Haramoto E., Kitajima M., Hata A., Torrey J.R., Masago Y., Sano D., Katayama H. (2018). A review on recent progress in the detection methods and prevalence of human enteric viruses in water. Water Res..

[88] Engvall E., Perlmann P. (1971). Enzyme-linked immunosorbent assay (ELISA). Quantitative assay of immunoglobulin G. Immunochemestry.

[89] Crowther J.R. (2009). Methods in Molecular Biology.

[90] Maier R.M., Pepper I.L., Gerba C.P. (2009). Enviromental Microbiology.

[91] He J., Wild D. (2013). Practical guide to ELISA development. The Immunoassay Handbook.

[92] Sakamoto S., Putalun W., Vimolmangkang S., Phoolcharoen W., Shoyama Y., Tanaka H., Morimoto S. (2018). Enzyme-linked immunosorbent assay for the quantitative/qualitative analysis of plant secondary metabolites. J. Nat. Med..

[93] McFeters G.A. (1990). Drinking Water Microbiology Progress and Recent Developements.

[94] Towbin H., Staehelin T., Gordon J. (1979). Electrophoretic transfer of proteins from polyacrylamide gels to nitrocellulose sheets: Procedure and some applications. Proc. Natl. Acad. Sci. USA.

[95] Bass J.J., Wilkinson D.J., Rankin D., Phillips B.E., Szewczyk N.J., Smith K., Atherton P.J. (2016). An overview of technical considerations for Western blotting applications to physiological research. Scand. J. Med. Sci. Sports.

[96] Jensen E.C. (2012). The basics of western blotting. Anat. Rec..

[97] Eslami A., Lujan J. (2010). Western blotting: Sample preparation to detection. J. Vis. Exp..

[98] Kurien B., Scofield R. (2006). Western blotting. Methods.

[99] Xu J., Sun H., Huang G., Liu G., Li Z., Yang H., Jin L., Cui X., Shi L., Ma T. (2019). A fixation method for the optimisation of western blotting. Sci. Rep..

[100] Meads M.B., Medveczky P.G., Specter S., Hodinka R., Young S., Wiedbrauk D. (2009). Application of western blotting to diagnosis of viral infections. Clinical Virology Manual.

[101] Mahmood T., Yang P.C. (2012). Western blot: Technique, theory, and trouble shooting. N. Am. J. Med. Sci..

[102] Taylor S.C., Berkelman T., Yadav G., Hammond M. (2013). A defined methodology for reliable quantification of western blot data. Mol. Biotechnol..

[103] La Rosa G., Muscillo M., Cook N. (2013). Molecular detection of viruses in water and sewage. Viruses in Food and Water: Risks, Surveillance and Control.

[104] Buzdin A.A., Buzdin A.A., Lukyanov S.A. (2007). Nucleic acids hybridization: Potentials and limitations. Nucleic Acids Hybridization Modern Applications.

[105] Mays Hoopes L.L., Gallagher S.R., Wiley E.A. (2008). Nucleic acid blotting: Southern and northern. Current Protocols Essential Laboratory Techniques.

[106] Yeh H.Y., Yates M.V., Chen W., Mulchandani A. (2009). Real-time molecular methods to detect infectious viruses. Semin. Cell. Dev. Biol..

[107] Girones R., Ferrús M.A., Alonso J.L., Rodriguez-Manzano J., Calgua B., Corrêa A.A., Hundesa A., Carratala A., Bofill-Mas S. (2010). Molecular detection of pathogens in water-the pros and cons of molecular techniques. Water Res..

[108] Van Pelt-Verkuil E., van Belkum A., Hays J.P., van Pelt-Verkuil E., van Belkum A., Hays J.P. (2008). The polymerase chain reaction. Principles and Technical Aspects of PCR Amplification.

[109] Ramírez-Castillo F.Y., Loera-Muro A., Jacques M., Garneau P., Avelar-González F., Harel J., Guerrero-Barrera A.L. (2015). Waterborne pathogens: Detection methods and challenges. Pathogens.

[110] Kadri K., Nagpal M.L., Boldura O.M., Balta C., Enany S. (2020). Polymerase chain reaction (PCR): Principle and applications. Synthetic Biology—New Interdisciplinary Science.

[111] Hryniszyn A., Skonieczna M., Wiszniowski J. (2013). Methods for detection of viruses in water and wastewater. Adv. Microbiol..

[112] Elnifro E.M., Ashshi A.M., Cooper R.J., Klapper P.E. (2000). Multiplex PCR: Optimization and application in diagnostic virology. Clin. Microbiol. Rev..

[113] Rodríguez R.A., Pepper I.L., Gerba C.P. (2009). Application of PCR-based methods to assess the infectivity of enteric viruses in environmental samples. Appl. Environ. Microbiol..

[114] Chen H., Larramendy M., Soloneski S. (2016). Nucleic acid detection of major foodborne viral pathogens: Human noroviruses and hepatitis A virus. Nucleic Acids—From Basic Aspects to Laboratory Tools.

[115] Kralik P., Ricchi M. (2017). A basic guide to real time PCR in microbial diagnostics: Definitions, parameters, and everything. Front. Microbiol..

[116] Srivastava K.R., Awasthi S., Mishra P.K., Srivastava P.K., Prasad M.N.V., Grobelak A. (2020). Biosensors/molecular tools for detection of waterborne pathogens. Waterborne Pathogens—Detection and Treatment.

[117] Watzinger F., Ebner K., Lion T. (2006). Detection and monitoring of virus infections by real-time PCR. Mol. Asp. Med..

[118] Singh J., Birbian N., Sinha S., Goswami A. (2014). A critical review on PCR, its types and applications. Int. J. Adv. Res. Biol. Sci..

[119] Wagner E.M. (2013). Monitoring gene expression: Quantitative real-time rt-PCR. Methods Mol. Biol..

[120] Hawkins S.F., Guest P.C. (2017). Multiplex analyses using real-time quantitative PCR. Methods Mol. Biol..

[121] Lievens A., Jacchia S., Kagkli D., Savini C., Querci M. (2016). Measuring digital PCR quality: Performance parameters and their optimization. PLoS ONE.

[122] Demeke T., Dobnik D. (2018). Critical assessment of digital PCR for the detection and quantification of genetically modified organisms. Anal. Bioanal. Chem..

[123] Neault N., Baig A.T., Graber T.E., D’Aoust P.M., Mercier E., Alexandrov I., Crosby D., Baird S., Mayne J., Pounds T. (2020). SARS-CoV-2 Protein in Wastewater Mirrors COVID-19 Prevalence. medRxiv.

[124] Ongerth J.E. (2020). RT qLAMP-Direct Detection of SARS-CoV-2 in Raw Sewage. medRxiv.

[125] Johnston J., Behrens S. (2020). Seasonal Dynamics of the Activated Sludge Microbiome in Sequencing Batch Reactors, Assessed Using 16S rRNA Transcript Amplicon Sequencing. Appl. Environ. Microbiol..

[126] Liu L., Li Y., Li S., Hu N., He Y., Pong R., Lin D., Lu L., Law M. (2012). Comparison of next-generation sequencing systems. J. Biomed. Biotechnol..

[127] Chan A.W., Naphtali J., Schellhorn H.E. (2019). High-throughput DNA sequencing technologies for water and wastewater analysis. Sci. Prog..

[128] Urban L., Holzer A., Baronas J.J., Hall M.B., Braeuninger-Weimer P., Scherm M.J., Kunz D.J., Perera S.N., Martin-Herranz D.E., Tipper E.T. (2021). Freshwater monitoring by nanopore sequencing. eLife.

[129] Acharya K., Blackburn A., Mohammed J., Haile A.T., Hiruy A.M., Werner D. (2020). Metagenomic water quality monitoring with a portable laboratory. Water Res..

[130] Kemp S., Collier D., Datir R., Ferreira I., Gayed S., Jahun A., Hosmillo M., Rees-Spear C., Mlcochova P., Lumb I.U. (2021). Neutralising antibodies in Spike mediated SARS-CoV-2 adaptation. Nature.

[131] Crits-Christoph A., Kantor R.S., Olm M.R., Whitney O.N., Al-Shayeb B., Lou Y.C., Flamholz A., Kennedy L.C., Greenwald H., Hinkle A. (2021). Genome sequencing of sewage detects regionally prevalent SARS-CoV-2 variants. mBio.

[132] Izquierdo-Lara R., Elsinga G., Heijnen L., Oude Munnink B.B., Schapendonk C.M.E., Nieuwenhuijse D., Kon M., Lu L., Aarestrup F.M., Lycett S. (2020). Monitoring SdumkARS-CoV-2 circulation and diversity through community wastewater sequencing. medRxiv.

[133] Jahn K., Dreifuss D., Topolsky I., Kull A., Ganesanandamoorthy P., Fernandez-Cassi X., Bänziger C., Stachler E., Fuhrmann L., Jablonski K.P. (2021). Detection of SARS-CoV-2 variants in Switzerland by genomic analysis of wastewater samples. Infect. Dis..

[134] Dumke R., de la Cruz Barron M., Oertel R., Helm B., Kallies R., Berendonk T.U., Dalpke A. (2021). Evaluation of two methods to concentrate SARS-CoV-2 from untreated wastewater. Pathogens.

[135] Bhalla N., Jolly P., Formisano N., Estrela P. (2016). Introduction to biosensors. Essays Biochem..

[136] Men D., Zhou J., Li W., Leng Y., Chen X., Tao S., Zhang X.E. (2016). Fluorescent Protein Nanowire-Mediated Protein Microarrays for Multiplexed and Highly Sensitive Pathogen Detection. ACS Appl. Mater. Interfaces.

[137] Yang Z., Anglès d‘Auriac M., Goggins S., Kasprzyk-Hordern B., Thomas K.V., Frost C.G., Estrela P. (2015). A novel DNA biosensor using a ferrocenyl intercalator applied to the potential detection of human population biomarkers in wastewater. Environ. Sci. Technol..

[138] Pilevar M., Kim K.T., Lee W.H. (2021). Recent advances in biosensors for detecting viruses in water and wastewater. J. Hazard. Mater..

[139] Velusamy V., Arshak K., Korostynska O., Oliwa K., Adley C. (2010). An overview of foodborne pathogen detection: In the perspective of biosensors. Biotechnol. Adv..

[140] Ahmed A., Rushworth J.V., Hirst N.A., Millner P.A. (2014). Biosensors for whole-cell bacterial detection. Clin. Microbiol. Rev..

[141] Mustafa F., Andreescu S. (2018). Chemical and biological sensors for food-quality monitoring and smart packaging. Foods.

[142] Ashrafi A.M., Koudelkova Z., Sedlackova E., Richtera L., Adam V. (2018). Electrochemical sensors and biosensors for determination of mercury ions. J. Electrochem. Soc..

[143] Peixoto A.C., Silva A.F., Rodrigues L., Mota M. (2017). 11—Smart devices: Micro- and nanosensors. Bioinspired Materials for Medical Applications.

[144] Thevenot D.R., Toth K., Durst R.A., Wilson G.S. (1999). Electrochemical biosensors: Recommended definitions and classification. Pure Appl. Chem..

[145] Ilkhani H., Farhad S. (2018). A novel electrochemical DNA biosensor for Ebola virus detection. Anal. Biochem..

[146] Saylan Y., Erdem O., Unal S., Denizli A. (2019). An Alternative Medical Diagnosis Method: Biosensors for Virus Detection. Biosensors.

[147] Freitas T.A., Proenca C.A., Baldo T.A., Materon E.M., Wong A., Magnani R.F., Faria R.C. (2019). Ultrasensitive immunoassay for detection of Citrus tristeza virus in citrus sample using disposable microfluidic electrochemical device. Talanta.

[148] Ozer T., Geiss B.J., Henry C.S. (2020). Chemical and Biological Sensors for Viral Detection. J. Electrochem. Soc..

[149] Han J.H., Lee D., Chew C.H.C., Kim T., Pak J.J. (2016). A multi-virus detectable microfluidic electrochemical immunosensor for simultaneous detection of H1N1, H5N1, and H7N9 virus using ZnO nanorods for sensitivity enhancement. Sens. Actuators B Chem..

[150] Kaya S.I., Karadurmus L., Ozcelikay G., Bakirhan N.K., Ozkan S.A., Han B., Tomer V.K., Nguyen T.A., Farmani A., Kumar Singh P. (2020). Electrochemical virus detections with nanobiosensors. Nanosensors for Smart Cities.

[151] Siuzdak K., Niedziałkowski P., Sobaszek M., Łęga T., Sawczak M., Czaczyk E., Dziabowska K., Ossowski T., Nidzworski D., Bogdanowicz R. (2019). Biomolecular influenza virus detection based on the electrochemical impedance spectroscopy using the nanocrystalline boron-doped diamond electrodes with covalently bound antibodies. Sens. Actuators B Chem..

[152] Anik Ü., Tepeli Y., Sayhi M., Nsiri J., Diouani M.F. (2018). Towards the electrochemical diagnostic of influenza virus: Development of a graphene–Au hybrid nanocomposite modified influenza virus biosensor based on neuraminidase activity. Analyst.

[153] Shariati M., Ghorbani M., Sasanpour P., Karimizefreh A. (2019). An ultrasensitive label free human papilloma virus DNA biosensor using gold nanotubes based on nanoporous polycarbonate in electrical alignment. Anal. Chim. Acta.

[154] Cabral-Miranda G., Cardoso A.R., Ferreira L.C.S., Sales M.G.F., Bachmann M.F. (2018). Biosensor-based selective detection of Zika virus specific antibodies in infected individuals. Biosens. Bioelectron..

[155] Palomar Q., Gondran C., Marks R., Cosnier S., Holzinger M. (2018). Impedimetric quantification of anti-dengue antibodies using functional carbon nanotube deposits validated with blood plasma assays. Electrochim. Acta.

[156] Lai H.C., Chin S.F., Pang S.C., Sum H., Sia M., Perera D. (2017). Carbon nanoparticles based electrochemical biosensor strip for detection of Japanese Encephalitis Virus. J. Nanomat..

[157] Lee T., Park S.Y., Jang H., Kim G.H., Lee Y., Park C., Mohsen M., Lee M.H., Min J. (2019). Fabrication of electrochemical biosensor consisted of multi-functional DNA structure/porous au nanoparticle for avian influenza virus (H5N1) in chicken serum. Mater. Sci. Eng. C.

[158] Zhang Y., Gao Y., Zhang X., Wang H., Xia T., Bian C., Liang S., Tng X., Wang X. (2019). Electrochemical immunosensor for HBe antigen detection based on a signal amplification strategy: The co-catalysis of horseradish peroxidase and nanoporous gold. Sens. Actuators B Chem..

[159] Hou Y.H., Wang J.J., Jiang Y.Z., Lv C., Xia L., Hong S.L., Lin M., Lin Y., Zhang Z.L., Pang D.W. (2018). A colorimetric and electrochemical immunosensor for point-of-care detection of enterovirus 71. Biosens. Bioelectron..

[160] Layqah L.A., Eissa S. (2019). An electrochemical immunosensor for the corona virus associated with the Middle East respiratory syndrome using an array of gold nanoparticle-modified carbon electrodes. Microchim. Acta.

[161] Sayhi M., Ouerghi O., Belgacem K., Arbi M., Tepeli Y., Ghram A., Anik U., Osterlunf L., Laouini D., Diouani M.F. (2018). Electrochemical detection of influenza virus H9N2 based on both immunomagnetic extraction and gold catalysis using an immobilization-free screen printed carbon microelectrode. Biosens. Bioelectron..

[162] Gao Z., Li Y., Zhang X., Feng J., Kong L., Wang P., Chen Z., Dong Y., Wei Q. (2018). Ultrasensitive electrochemical immunosensor for quantitative detection of HBeAg using Au@Pd/MoS2@MWCNTs nanocomposite as enzyme-mimetic labels. Biosens. Bioelectron..

[163] Tu H., Lin K., Lun Y., Yu L. (2018). Magnetic bead/capture DNA/glucose-loaded nanoliposomes for amplifying the glucometer signal in the rapid screening of hepatitis C virus RNA. Anal. Bioanal. Chem..

[164] Ganser L.R., Kelly M.L., Herschlag D., Al-Hashimi H.M. (2019). The roles of structural dynamics in the cellular functions of RNAs. Nat. Rev. Mol. Cell Biol..

[165] Zhang H., Miller B.L. (2019). Immunosensor-based label-free and multiplex detection of influenza viruses: State of the art. Biosens. Bioelectron..

[166] Xiao T., Huang J., Wang D., Meng T., Yang X. (2020). Au and Au-Based nanomaterials: Synthesis and recent progress in electrochemical sensor applications. Talanta.

[167] Faria H.A.M., Zucolotto V. (2019). Label-free electrochemical DNA biosensor for zika virus identification. Biosens. Bioelectron..

[168] Khater M., de la Escosura-Muñiz A., Quesada-González D., Merkoçi A. (2019). Electrochemical detection of plant virus using gold nanoparticle-modified electrodes. Anal. Chim. Acta.

[169] Malik A.A., Nantasenamat C., Piacham T. (2017). Molecularly imprinted polymer for human viral pathogen detection. Mater. Sci. Eng. C.

[170] Babamiri B., Salimi A., Hallaj R. (2018). A molecularly imprinted electrochemiluminescence sensor for ultrasensitive HIV-1 gene detection using EuS nanocrystals as luminophore. Biosens. Bioelectron..

[171] Wangchareansak T., Thitithanyanont A., Chuakheaw D., Gleeson M.P., Lieberzeit P.A., Sangma C. (2013). Influenza A virus molecularly imprinted polymers and their application in virus sub-type classification. J. Mater. Chem. B.

[172] Wangchareansak T., Thitithanyanont A., Chuakheaw D., Gleeson M.P., Lieberzeit P.A., Sangma C. (2014). A novel approach to identify molecular binding to the influenza virus H5N1: Screening using molecularly imprinted polymers (MIPs). MedChemComm.

[173] Tai D.F., Lin C.Y., Wu T.Z., Chen L.K. (2005). Recognition of dengue virus protein using epitope-mediated molecularly imprinted film. Anal. Chem..

[174] Altintas Z., Pocock J., Thompson K.A., Tothill I.E. (2015). Comparative investigations for adenovirus recognition and quantification: Plastic or natural antibodies?. Biosens. Bioelectron..

[175] Jenik M., Schirhagl R., Schirk C., Hayden O., Lieberzeit P., Blaas D., Paul G. (2009). Sensing picornaviruses using molecular imprinting techniques on a quartz crystal microbalance. Anal. Chem..

[176] Lodder W., de Roda Husman A.M. (2020). SARS-CoV-2 in wastewater: Potential health risk, but also data source. Lancet Gastroenterol. Hepatol..

[177] Mao K., Zhang H., Yang Z. (2020). Can a Paper-Based Device Trace COVID-19 Sources with Wastewater-Based Epidemiology?. Environ. Sci. Technol..

[178] Klug K.E., Reynolds K.A., Yoon J.Y. (2018). A Capillary Flow Dynamics-Based Sensing Modality for Direct Environmental Pathogen Monitoring. Chem. Eur. J..

[179] McCracken K.E., Tat T., Paz V., Reynolds K.A., Yoon J.Y. (2017). Immunoagglutinated particle rheology sensing on a microfluidic paper-based analytical device for pathogen detection. ASABE Annual International Meeting.

